# For Whom the Price Escalates: High Price and Uncertain Value of Cancer Drugs

**DOI:** 10.3390/ijerph19074204

**Published:** 2022-04-01

**Authors:** Gyeongseon Shin, Hye-Young Kwon, SeungJin Bae

**Affiliations:** 1 College of Pharmacy, Ewha Womans University, Seoul 03760, Korea; sunny628@g.ewha.ac.kr; 2 Division of Biology and Public Health, Mokwon University, Deajeon 35349, Korea; haeyoungkwon0111@gmail.com

**Keywords:** cancer drug, oncology drug, value, expedited review, economic evaluation

## Abstract

The price of cancer drugs has skyrocketed, yet it is not clear whether their value is commensurate with their price. More cancer drugs are approved under expedited review, which considers less rigorous clinical evidence, yet only 20% of them show an overall survival gain in the confirmatory trial. Moreover, clinical data are often generated based on small, single-arm studies with surrogate outcomes, challenging economic evaluation. With their high price and uncertain (marginal) clinical value, cancer drugs are frequently rejected by health technology assessment (HTA) bodies. Therefore, agencies, including the UK’s National Institute for Health and Care Excellence (NICE), have adopted cancer drug funds (CDF) or risk-sharing schemes to provide extra access for expensive cancer drugs which fail to meet NICE’s cost effectiveness threshold. With rising pricing and fewer new cancer medications with novel mechanisms of action, it is unclear if newly marketed cancer therapies address unmet clinical needs or whether we are paying too much. Transparency, equity, innovativeness, and sustainability are all harmed by a “special” approach for cancer medications. If early access is allowed, confirmatory trials within a certain time frame and economic evaluation should be conducted, and label changes or disinvestment should be carried out based on those evaluations.

## 1. Introduction

Global spending on cancer drugs rose to USD 164 billion in 2020, with the average annual growth rate being 14.3% over the past 5 years [1]. For drugs approved by the US Food and Drug Administration (FDA) in 2018, the average price per patient per course was USD 150,384 [2] and is expected to increase even further with the introduction of more advanced treatment options (such as chimeric antigen receptor (CAR)-engineered T cell (CAR-T cell) therapy). The financial toxicity of expensive cancer drugs and concerns about affordability and sustainability have been raised in many developed and developing countries [3,4,5,6], while patients have spoken out in favor of quick access to cancer drugs [7]. 

Yet, it is not clear whether the clinical benefit of cancer drugs corresponds to their high price. The average incremental gain in the overall survival (OS) of the new cancer drugs approved by the FDA and European Medicine Agency (EMA) between 2003 and 2013 was 3.43 months [8]. In Europe, Australia, and the United States, the price of cancer drugs was weakly [9] or not significantly [10,11] associated with their clinical benefit [12]. However, their clinical and economic value is not rigorously examined, such as by expediting regulatory review or waiving economic evaluation. 

In general, a rigorous review process of efficacy and side effects is applied to drug approval. Following the thalidomide tragedy in 1962, which was shown to cause severe congenital disorders, the United States Congress legally required evidence of efficacy before approving a drug through the Kefauver–Harris Drug Amendments to the Federal Food, Drug, and Cosmetic Act (FDCA) [13]. This thorough process extended FDA review times to more than 30 months in the early 1980s, and manufacturers and industry organizations voiced worry that it was taking too long to approve lifesaving treatments. Since then, the approval and regulatory process has been added to special programs such as the Orphan Drug Act in 1983, expanded access in 1987, Fast-Track in 1988, priority review and Accelerated Approval in 1992, and breakthrough therapy designation in 2012. As a result, the proportion of cancer drugs being approved under the expedited approval program is increasing [14,15]. 

However, this might allow the approval of drugs based on less rigorous evidence, such as unvalidated surrogate outcomes or a single arm [16,17,18]. The improvement in the surrogate outcomes was frequently not verified to improve overall survival in the confirmatory trials or meta-analysis [19,20]. Moreover, 40% of oncology drugs with expedited review have not completed confirmatory trials or have not demonstrated their benefits, raising concern regarding their efficacy [14]. Of the cancer drugs that have received accelerated approval thus far, 17 have been either withdrawn by the FDA (e.g., bevacizumab for HER2-negative breast cancer) or voluntarily withdrawn by the manufacturer (e.g., nivolumab and pembrolizumab for small cell lung cancer (SCLC)) after failing to prove a clinical benefit in the post-approval trial [21,22].

Since clinical evidence of cancer drugs is often generated based on small, single-arm studies with surrogate outcomes, conducting economic evaluation for those cancer drugs is challenging [23]. With their high prices and uncertain (marginal) clinical value, cancer drugs are frequently rejected by health technology assessment (HTA) bodies. As a result, countries such as the United Kingdom and Canada have adopted separate processes, such as cancer drug funds (CDF), risk-sharing schemes, and pan-Canadian Oncology Drug Review (pCODR), to provide extra access for expensive cancer drugs that failed to meet the cost effectiveness threshold [24]. 

Then, do the “special” paths help cancer patients’ health, encourage the development of innovative drugs, and maximize population health, besides their high price? Maybe not. If cancer drugs without rigorous clinical evidence can be approved and reimbursed despite their high price, then patients may take cancer drugs with no clear benefit or that even cause potential risks, which may hurt patients’ health. Drugs reimbursed based on special pathways, such as CDF, have a poor cost effectiveness ratio and uncertain clinical value. Therefore, the need for those separate processes has been questioned in terms of value, fairness, and transparency [25,26]. With their prices being so high, easy access also harms the sustainability of the health care budget [2]. 

Moreover, it signals to pharmaceutical industries that less innovative drugs (second in class) with uncertain clinical value can still bring financial success [27]. The recent trend shows that the proportion of drugs with new mechanisms of action has decreased [28], suggesting newly developed drugs may not address unmet clinical needs while their prices have soared. Yet, cancer drugs have comprised the highest revenue of pharmaceuticals over the last decade [27], with global spending expected to reach USD 269 billion by 2025 [1]. Although accessing drugs with uncertain clinical and economic value may allow patients to have early access to drugs, it also creates several problems that we should carefully consider.

## 2. Proposals

Is it worth it to have a separate process just for cancer? Each society may have different preferences, yet a “special process” should be cautiously adopted only after an active discussion on the pros and cons. Having early access to drugs, although the drugs have uncertain clinical value, might be the only option for some patients without any alternatives. However, it hurts not only equity, transparency, innovation, and sustainability but also potentially patients’ health since the risk is not rigorously reviewed [29]. 

**Figure 1:**
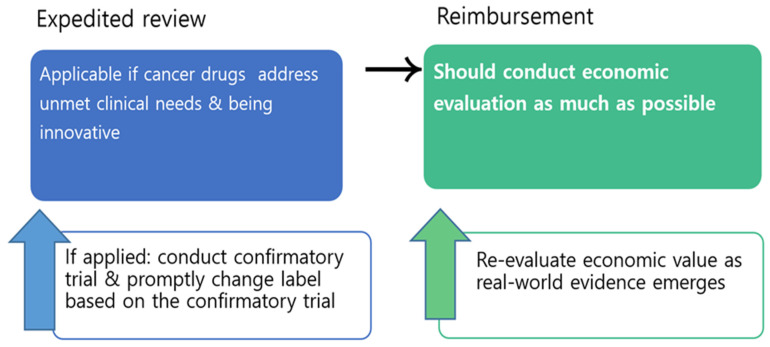
Market access process proposal for cancer drugs.

Thus, a special process should be applied only in limited conditions (Does it address unmet clinical needs? Is it innovative?), and if expedited review is applied to certain drugs, then their clinical and economic evidence should be routinely reviewed and appropriate action should be taken. For example, confirmatory clinical trials should be conducted for all cancer drugs approved under expedited review, and an immediate change of label should be followed if the promised outcome was not validated in the confirmatory trial. The regulatory agencies should be more active and even aggressive in compelling confirmatory trials within a certain time frame and promptly change labels based on the trials. In addition, although challenging, economic evaluation of cancer drugs should be conducted as much as possible, and the value of the cancer drugs should be re-evaluated as real-world evidence emerges, thus giving the industry a signal that cancer drugs with uncertain values will be eventually evaluated as such. Having a separate fund for cancer, such as CDF, is not sustainable, lacks justification, and is not fair. Instead of waiving economic evaluation for cancer drugs, flexible application of the threshold for cancer drugs, within the national health system, should be implemented (Figure 1). 

Along with the aging population, the burden of cancer will escalate, and the challenges related to expensive cancer drugs will intensify. In the face of soaring cancer drug expenditure, we need to clarify boundaries and send clear signals to industries before it is too late.

## Data Availability

Not applicable.
